# Influence of Resource Availability on the Foraging Strategies of the Triangle Butterflyfish *Chaetodon triangulum* in the Maldives

**DOI:** 10.1371/journal.pone.0151923

**Published:** 2016-03-21

**Authors:** Josie F. Chandler, Deborah Burn, Per Berggren, Michael J. Sweet

**Affiliations:** 1 School of Marine Science & Technology, University of Newcastle upon Tyne, Newcastle upon Tyne, Tyne and Wear, United Kingdom; 2 Environmental Sustainability Research Centre, University of Derby, Derby, United Kingdom; Hokkaido University, JAPAN

## Abstract

Obligate coral feeders such as many members of the Chaetodontidae family (also known as butterflyfish) often show strong preferences for particular coral species. This is thought to have evolved through natural selection as an energy-maximising strategy. Although some species remain as highly specialised feeders throughout their lifetime, many corallivores show a degree of dietary versatility when food abundance is limited; a strategy described by the optimal foraging theory. This study aimed to examine if, within-reef differences in the feeding regime and territory size of the Triangle Butterflyfish *Chaetodon triangulum* occurred, as a function of resource availability. Results showed that the dietary specialisation of *C. triangulum* was significant in both areas of low and high coral cover (χL2^2^ = 2.52 x 102, P<0.001 and χL2^2^ = 3.78 x 102, P<0.001 respectively). Resource selection functions (RSFs), calculated for the two main sites of contrasting coral assemblage, showed that in the resource-rich environments, only two Genera (*Acropora* and *Pocillopora*) were preferentially selected for, with the majority of other corals being actively ‘avoided’. Conversely, in territories of lower coral coverage, *C. triangulum* was being less selective in its prey choice and consuming corals in a more even distribution with respect to their availability. Interestingly, coral cover appeared to show no significant effect on feeding rate, however it was a primary determinant of territory size. The findings of the study agree with the predictions of the optimal foraging theory, in that where food supply is scarce, dietary specialisation is minimised and territory size increased. This results in maximising energy intake. This study represents the first scientific evidence that *C. triangulum* is an obligate corallivore and, as with many other butterflyfish, is therefore dependent on healthy scleractinian corals for survival.

## Introduction

Foraging strategies that enable a species to respond to the changing environment will yield greater benefits for their survival and reproductive output than specialisation [1]. The optimal foraging theory (OFT), first described by Macarthur and Pianka in 1969 [2], attempts to explain resource use and dietary patterns in animals. Later used in a marine context by Hughes in 1980 [3], OFT describes foraging behaviour as decisions made by an organism to maximise energy input per unit of effort [4]. The theory predicts that when an optimal resource is abundant, i.e. a prey item that provides the highest energy return, then that prey would be exclusively consumed as the forager can afford to bypass substandard prey items due to the high likelihood of encountering more profitable ones [5]. However, in conditions where preferred prey are not sufficiently available, shifts in prey selectivity should then occur and broader diets of less-preferred prey would be consumed in order to meet basic metabolic needs with minimum energy expended searching [6, 7].

Obligate coral feeders such as many members of the Chaetodontidae family (also known as butterflyfish) are constrained by the availability of corals, as these form a major component of their diet. As a means of enhancing fitness by maximising energy acquisition, some butterflyfish (e.g. *Chaetodon trifascialis*) have evolved to be specialists [8]. Many researchers describe extreme dietary specialisation as a highly risky strategy [5, 7, 9], which increases the species’ vulnerability to resource depletion. Although reports have shown that when specialist species inhabit resource-rich environments they are capable of outcompeting generalist congenerics [5], manipulative field studies record specialists as sustaining dramatic reductions in body weight and hepatic and gonad condition when only non-preferred prey are available [7].

For animals that maintain feeding territories, again as many butterflyfish species do, OFT may also account for variation in territory size and the extent of their territory defence [1, 10]. The theory predicts a complex trade-off between the energy expenditure required to defend a territory and the profitability of exclusive access to the resources within it, this in turn defines the size of territories and determines whether aggressive territory defence is economically viable [1]. For many reef dwelling organisms, percentage live coral cover has, unsurprisingly, been recognised as an important determinant of territory size [10], this is together with the composition of coral species within any given site [11]. For example, Gassner et al. [12] found a strong inverse correlation between percentage of living coral cover and territory size, aggressive encounters and feeding rates of the Blacktail butterflyfish *Chaetodon austriacus*.

Although there are many studies which evaluate the feeding regimes of butterflyfish, no studies have focused specifically on the feeding behaviour of butterflyfish in the Indian Ocean, despite this region being of tremendous biodiversity importance. Furthermore, there are certain species such as the Triangle butterflyfish, *Chaetodon triangulum* in which data is lacking for their specific association with coral. In fact, for this species there is only anecdotal reports of its feeding behaviour [8, 13, 14]. The sister species, the Eastern Triangle butterflyfish *Chaetodon baronessa*, has, in contrast, been well studied, with evidence highlighting a preference for *Acroporas* throughout their range [1, 15, 16].

The purpose of this study was to investigate the feeding behaviour of *C*. *triangulum* at Vavvaru Island in the Maldives. At this study location coral coverage and composition is variable between sites allowing for a comparative study to be undertaken on a small spatial scale. The study assessed whether the foraging behaviour of *C*. *triangulum* conforms to the predictions of OFT in contrasting coral habitats and we set out to answer; 1) how does scleractinian coral dietary specialisation of *C*. *triangulum* vary with changes in coral assemblage? and; 2) is there a significant correlation between percentage live coral cover and feeding rate and/or territory size in *C*. *triangulum*? Furthermore, by quantifying the dietary specialisation of *C*. *triangulum* and the degree to which it relies on particular corals, we would be able to give an indication of how vulnerable this species may be with relation to the threat of coral depletion.

## Materials and Methods

### Study Site

The study was carried out on the eastern side of Vavvaru Island (5.4177° N, 73.3547°E) located in Lhaviyani Atoll, North Province, Maldives. Data collection was conducted between June and October 2013. The Ministry of Fisheries and Agriculture, Maldives issued the permission for conducting marine scientific research at this location. Specific permissions were not required for the activities as they were observational and did not involve endangered or protected species.

Sampling was conducted on the contiguous shallow reef flats and reef crests (1-4m depth) situated on the sheltered lagoon-facing shore. All surveys were carried out by snorkelling at a distance of 1-3m from the reef. The study species, *Chaetodon triangulum* (Triangle butterflyfish), is common and widespread throughout the Indian Ocean. It is known to be a monogamous species [17] which will actively defend its feeding territory as a pair. *C*.*triangulum* territories were selected prior to the start of the study by random swimming over the chosen site area. Eight territories of low coral cover (<25% live coral) on the more exposed, south-western side of the reef and nine of high coral cover (>40%) on the sheltered eastern side were surveyed during the study for comparisons of optimal foraging behaviour, data was pooled for each of the two sites. To ensure data at each site was suitable for analysing separately, an un-paired t-test was performed with 95% confidence intervals and this clarified a significant difference between the two sample means. The data conformed to all assumptions for the t-test (Low cover: Kolmogorov-Smirnov, P>0.05 and High cover: Kolmogorov, P>0.05; F-test, F = 0.56, P>0.05). There was a significant difference between mean live coral coverage for ‘Low Cover’ sites (mean = 15.57 ± 1.87% S.E.) and ‘High Cover’ sites (mean = 49.44 ± 2.34% S.E.) (t-test, t = -11.02 df = 15, *P* < 0.001).

### Territories

The term ‘territory’ is disputed in the literature with authors claiming agonistic behaviour must be present for a home range to be termed a territory [18]. During our study, *C*. *triangulum* was observed actively chasing congenerics away from coral resources and their monogamous mate, we therefore use the term territory to describe the actively defended observed foraging home range.

A total of 23 focal pairs of *C*. *triangulum* were randomly selected for observations of foraging and spatial behaviour. For assessing *C*. *triangulum* home ranges, pairs were observed for 30 minutes and coloured markers were dropped and moved to fit the boundary of the fishes’ movements. Note; the markers were dropped after the fish had moved away from the boundary in order to minimise any impact on the fish’s movements. Measurements were taken of each territory perimeter [10]. In addition to the eight territories of low coral cover and the nine territories of high coral cover, a further six territories consisting of intermediate coral cover (26–39%) were added to the dataset for this correlation aspect of the study. All territories were non-overlapping.

### Feeding Observations

Feeding observations were carried out on one focal pair within each territory to assess dietary composition. The range of prey types eaten as well as their proportional consumption was recorded during ten consecutive 3-minute observation periods [19]. Qualitative descriptions on foraging behaviour such as repetitive biting at single coral heads and extended swimming periods were also recorded. As gender was difficult to ascertain in the field, observations alternated between individuals of the focal pair to minimise any possible impacts gender may have caused on feeding behaviour [8].

For each feeding observation, the scleractinian corals which were fed upon were categorised to genera using Veron’s *Corals of the World* books [20] and any other non-coral prey were also recorded. Identification was not conducted to species level during this study, however future studies should consider this to highlight if feeding preferences occur within genera as well as between them.

Analogous to other studies, most fish continued to feed despite the presence of the observer, but in any case where fish fled or sought shelter, observations were discontinued [16, 19]. As the majority of butterflyfish are diurnally active [21–23], feeding observations were conducted during daylight hours, consistently between 10:00hrs and 14:00hrs. Overall, 500+ minutes of feeding behaviour observations were carried out.

### Resource Availability

Variation in the availability of coral prey was assessed by surveying transects within the fish territories. A 1 x 50m transect tape was laid randomly through the fish territories and photographs were taken of the substrate directly below the tape at 1m intervals. A Nikon Coolpix AW110 waterproof camera was used for photographing the substrate and a monopod camera apparatus, consisting of a 70cm pole supporting the camera, ensured precision and consistency of the frame size (50x60cm) which ensured the photographs were non-overlapping. Ten random points were generated within each photograph using PhotoGrid 1.0 software and the substrate directly beneath each of the ten sampling points was recorded. Scleractinian corals were identified to genera (as above), while other substrate types were categorised to one of six categories (soft coral, sponge, macroalgae, sand, rubble or rock). The majority of bites during the feeding observations were on a small sub-set of six different coral genera (*Acropora*, *Pocillopora*, *Porites*, *Fungia*, *Galaxea and Montipora*). Corals including *Leptoria*, *Pavona*, *Favia*, *Favites*, *Lobophyllia and Pachyseris* were also fed upon but less frequently; these were grouped with unidentified scleractinian corals to form an ‘Other Coral’ category. The percentage live coral cover and overall composition of scleractinian corals was calculated from their proportion of the 500 points per territory.

### Dietary Specialisation and Selectivity

Dietary specialisation was investigated using the *χ*^2^ log-likelihood statistic [24], following Manly et al’s Design II and Sampling Protocol A. This approach has been used by Pratchett [15] and Lawton et al. [25]. The statistic tests for selectivity among all the resources. *χ*_L2_^2^ was calculated using the formula below and then compared to the *χ*^2^ distribution to establish the significance of the dietary selectivity exhibited at each site:

$${\chi L2}^{2}=2\sum_{j=1}^{n}\sum_{i=1}^{i}u_{ij}ln\left\{u_{ij}/E\left(u_{ij}\right)\right\}$$

Where u_ij_ is the proportional use of each prey type (*i*) by each individual (*j*) and E(u_*ij*_) is the expected number of bites taken from prey type *i* by the individual *j* if use is proportional to availability [26]. *χ*_L2_^2^ was calculated for both low and high cover sites. Higher values of *χ*_L2_^2^ indicate increasing specialisation.

Resource selection functions (RSFs) were calculated which allow for the assessment of an animal’s feeding selectivity with a use versus availability framework. We utilised them in this study to establish which coral genera were being used disproportionately to their availability. Manly’s RSF [26] was credited as the preferred electivity index in a recent review paper by Lawton et al. [25], the function gives an indication of where prey preferences lie and can be used to assess an animal’s level of dependence on specific resources.

RSF was calculated using the formula;

$$w_{i}=\left\{\sum_{j=1}^{n}u_{ij}/p_{i}\right\}/n$$

Where u_*ij*_ is the proportional use of prey category *i* by the *j*th individual, p_*i*_ is the proportional availability of each prey type within each site and *n* is the number of individuals sampled [26]. Resulting values higher than 1 indicate preference, values lower than 1 indicate avoidance. Bonferroni corrected 95% confidence intervals calculated around the resource selection functions provide a statistical basis for the electivity analysis.

### Data Analysis

Data analysis was carried out in Minitab 16. For relationships between territory size and other variables, scatter plots were drawn and correlation coefficients calculated. Regression analysis was used to derive equations for the fitted line plots. Living coral cover data conformed to normality (Kolmogorov-Smirnov, P>0.05), however data for territory size did not, therefore data were log^10^ transformed. The Method of Least Squares Regression was used to derive the equation for the fitted line. Log^10^ transformation of the data had little effect on the fit of the line (log^10^ transformed = P<0.001, r-sq: 58.5%). Data on feeding rates were checked for normality but were found not to conform to these assumptions (Kolmogorov-Smirnov, P<0.05). Data were ranked and Spearman’s correlation coefficient was calculated for the relationship between feeding rate and live coral cover.

## Results

### Dietary Composition and Selectivity

#### Overall Feeding Behaviour

**Fig 1:**
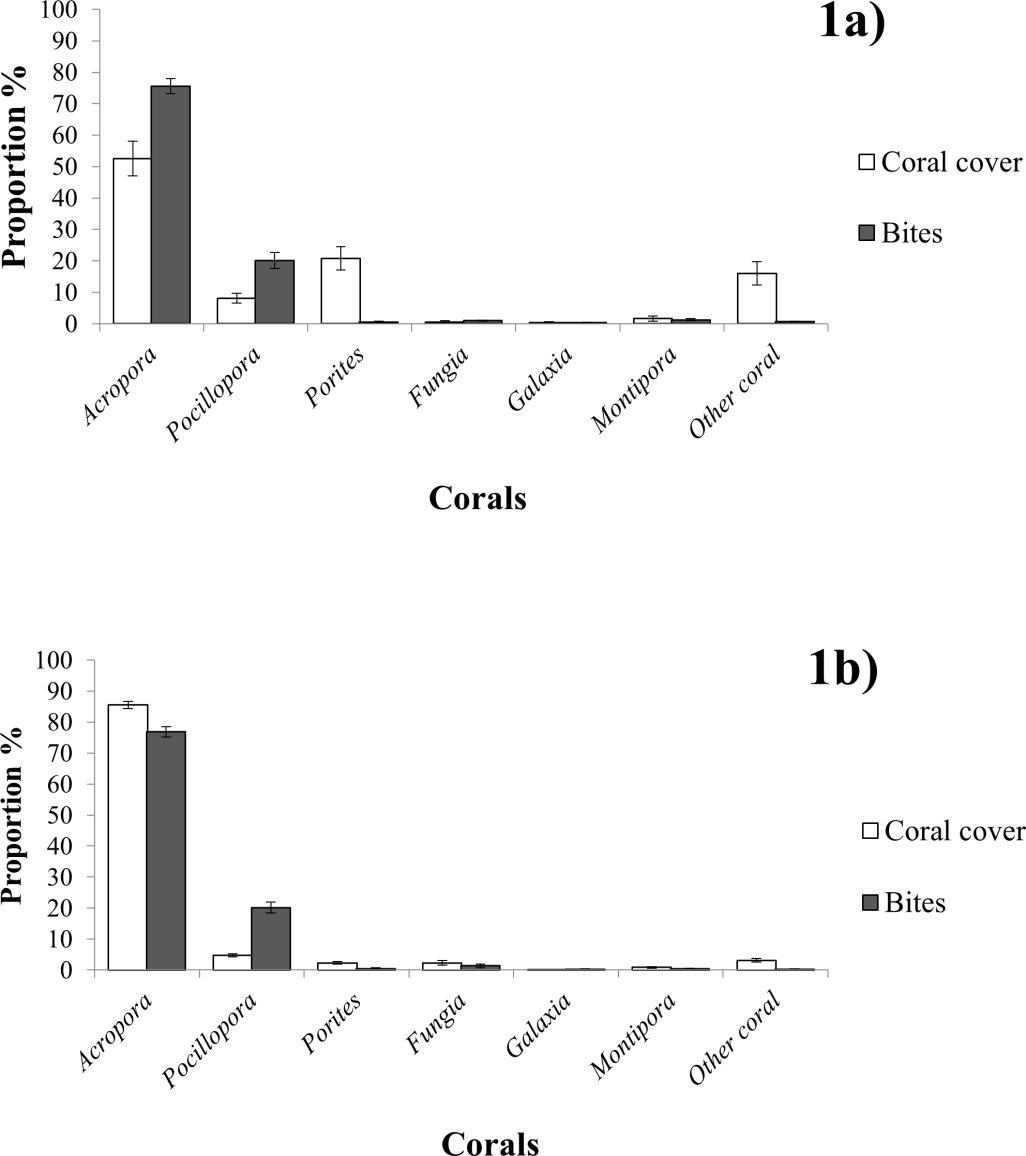
**Coral cover and proportional consumption, compared at sites of different coral coverage** Mean ± S.E. proportional cover of scleractinian corals and mean ± S.E. proportion of bites taken on respective corals during feeding observations of *Chaetodon triangulum* at sites of (**a)** low coral coverage (<25%) (n = 8 territories) and (**b)** high coral coverage (>40%) (n = 9 territories) in the Maldives, June-October 2013.

*Chaetodon triangulum* fed almost exclusively on live scleractinian corals (Fig 1A and 1B) with the exception of three occasions where individuals were observed to feed on dead *Acropora* or the mucus of bleached corals (not included in the data). *χ*^2^ Log likelihood analysis was carried out on pooled data for Low Cover and High Cover territories and indicated that in both conditions *C*. *triangulum* exhibited significant selectivity in its patterns of feeding (P<0.001, Table 1). Higher values of *χ*_*L2*_^2^ were determined for feeding at the High Cover sites (Table 1), suggesting that specialisation was stronger for individuals within these resource-rich territories. Selection functions greater than 1.00 indicate that corals are being used disproportionately more than expected from their availability, therefore denoting a preference, whilst selection functions less than one indicate that corals are being consumed less than expected, denoting prey types that are being avoided [26]. No significant correlation was determined between availability of each coral genus and the proportion of bites on that coral (Spearman Rank Correlation Coefficient, n = 23, P>0.05).

**Table 1:** Dietary selectivity and prey preferences of *Chaetodon triangulum* presented as chi-square log likelihood analysis (*χ*_*L2*_^2^) and resource selection functions for seven coral categories [26]. ‘Low’ indicates pooled data for sites with <25% living coral coverage and ‘High’ indicates pooled data for sites with >40% living coral coverage.

	*n*	*χ*_*L2*_^2^	*P*	*Acropora*	*Pocillopora*	*Porites*	*Fungia*	*Galaxea*	*Montipora*	Other coral
Low	8	2.52 x 10^2^	<0.001	1.29	2.47	0.43	0.47	0.00	0.16	0.31
High	9	3.78 x 10^2^	<0.001	0.90	4.38	0.63	1.57	0.72	0.58	0.18

### Coral Composition, Diet and Selectivity at Low Cover Sites

Percentage live coral cover from the low cover’ sites had an average of 15.6 ± 1.9% (mean ± S.E.). *Acropora* was much less dominant within low cover territories than it was within high cover territories. In total, *Acropora* made up 53.9 ± 5.0% (mean ± S.E.) of the live coral cover (Fig 1A) at low cover sites comprising only 9.0 ± 1.8% (mean ± S.E.) of the total substrate within these sites. In contrast, at high cover sites *Acropora* accounted for 85.5 ± 3.27% (mean ± S.E.) of the live coral cover and 42.3 ± 2.1% (mean ± S.E.) of total substrate.

Resource selection functions for *Acropora* and *Pocillopora* (Table 1) demonstrate that *C*. *triangulum* exhibited significant selectivity for these genera; both were grazed disproportionately more than would be expected from their relative availability (Fig 1A). Although both coral genera showed such a trend, the higher selection function for *Pocillopora* indicates that the strongest selection was for this genera.

The selection functions calculated for *Fungia* and *Galaxea* gave results lower than 1 (Table 1), suggesting that *C*. *triangulum* was actively avoiding these corals. These genera were being grazed in approximate accordance to their availability (low substrate coverage, Fig 1A) but the presence of some anomalous zero values in these samples have resulted in selection functions that are closer to 0 than 1. This is a limitation of the electivity index as it constructs a value of selectivity from an average of individuals, thus creating unrepresentative results if there are anomalies in the data set.

*Porites* and those classed as ‘Other Coral’ were two of the most common substrate groups in the low cover territories (Fig 1A), however the consumption of these corals was relatively rare. Regardless of rarity, selectivity analysis consequently indicated a strong avoidance of these genera (Table 1).

### Coral Composition, Diet and Selectivity at High Cover Sites

Percentage live coral cover in the high cover sites ranged from 40.0% to 61.4% with a mean of 49.4 ± 2.3% (mean ± S.E.). *Acropora* species dominated the benthos of these territories. Feeding on *Acropora* species accounted for 77 ± 1.6% (mean ± S.E.) of all bites during the observations, however these corals appeared to not be selected for (Table 1), due to their roughly proportional availability (85 ± 1.2% S.E.) (Fig 1B). *Pocillopora* in contrast was consumed in excess of its abundance (approximately four times that of its proportional availability (Fig 1B), resulting in the highest mean selection function (Table 1) and an indication of significant dietary selectivity for this genera. The relative abundances of *Porites* (2.3 ± 0.4% S.E.) and ‘Other Coral’ (3.4 ± 2.0% S.E.) were much lower in these territories than they were in the low cover sites. Interestingly, selection functions suggested *C*. *triangulum* may also preferentially select *Fungia* in high cover sites (Table 1), however this result might have resulted from the abnormally high feeding count within one of the territories, thereby skewing the statistic.

### Feeding Rates

No statistically significant relationship between mean feeding rate of *C*. *triangulum* (number of bites per 3 minutes) and percentage live coral cover was found (Spearman Rank Correlation Coefficient, r_s_ = -0.128, n = 23, P>0.05).

### Territory Size

**Fig 2:**
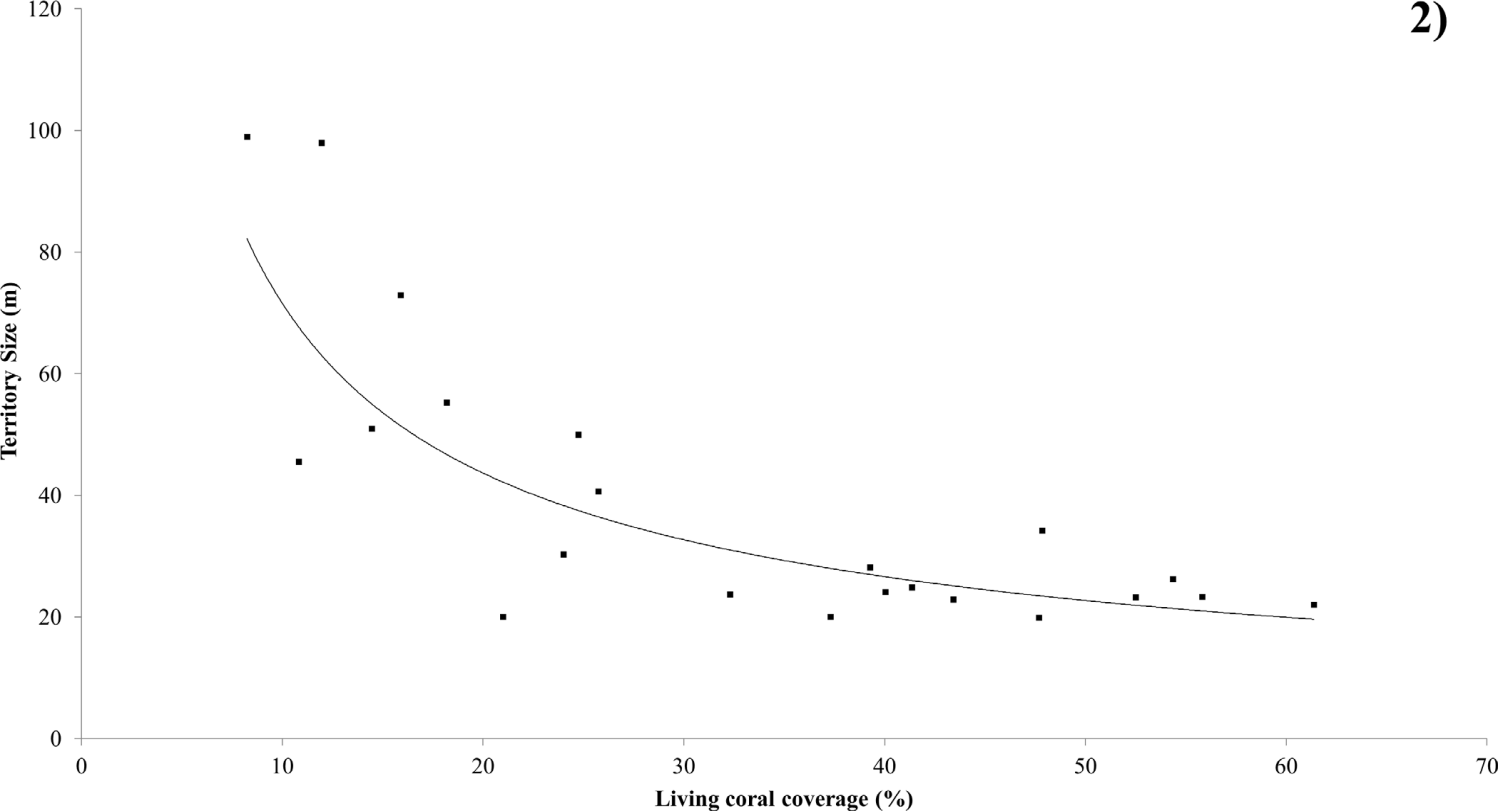
Relationship between coral cover and territory size. Effect of living coral coverage on the territory size of *Chaetodon triangulum*. The fitted line is described by the equation y = 369.76x^-0.713^; R^2^ = 0.690 (P<0.05).

Territory size (perimeter) ranged from 19.9m to 99m across all sites (Fig 2). When data from all 23 *C*. *triangulum* pairs were pooled, there was a negative correlation between living coral cover and perimeter of territory (Spearman Rank Correlation Coefficient rs = -0.694, n = 22, P<0.001) (Fig 2).

There was a significant relationship determined between living coral cover and territory size (Regression ANOVA: F = 10.98, df = 1, P<0.001). The power regression explains 75.93% of variation. There was no significant correlation between feeding rate and territory size (Spearman Rank Correlation Coefficient, rs = 0.347, n = 23, P = 0.114).

## Discussion

*Chaetodon triangulum*, *Chaetodon baronessa* and many other territory-defending fish including damselfish [27] have all demonstrated inverse relationships between territory size and coral abundance which are consistent with predictions of optimal foraging. Increases in territory size have been ascribed as a compensatory response to the reduced food access [4]; expanding ranges reflects the fishes’ need to forage over a larger area in order to obtain sufficient nutrients for the pair [1], territory defence is also predicted to play a role. Interestingly, in this study, we found that feeding rates of *C*. *triangulum* appeared to not show any significant relationship to live coral cover or territory size. Such a result may have occurred as rapid feeding bouts on individual coral heads followed by long search times were observed, which may be a specific energy maximising strategy by *C*. *triangulum*, however this behaviour was not represented in the data on ‘mean feeding rate’. In future studies we suggest that the feeding strategy of such fish would be better represented by recording activity budgets. It is likely the ‘search time’ budget for fish in low cover sites would have been significantly longer than of those in high cover sites if this had been assessed.

Fish are defined as obligate corallivores if scleractinian corals comprise more than 80% of their diet [8]. The findings of this study provide strong evidence that *C*. *triangulum* belongs to this category of fish, having fed almost exclusively upon hermatypic corals for the entirety of the study, with infrequent exceptions being bites on the nutrient-rich mucus of bleached corals. Furthermore, this study shows that *C*. *triangulum* is a ‘functional specialist’, as described by Cole *et al*. [8], whereby it has obvious prey preferences but these selective feeding habits give way to dietary versatility in areas where available food is scarce. Corals from the genus *Acropora* were shown to make up a major component of *C*. *triangulum’s* diet however it was *Pocillopora* species that appear to be the preferred choice for *C*. *triangulum* where possible (Table 1). It should be noted that for studies such as this, identification of corals to species level would have been preferable, however with such high diversity found in the Indian Ocean this is often problematic and therefore identification to genus level was used. Results from this study suggest that we should, at the very least, explore different species of both *Acopora* and *Pocillopora* to see if there is a species preference within these genera for *C*. *triangulum*. Regardless, such opportunistic exploitation of preferred dietary resources, as shown in this study, highlight that *C*. *triangulum* conforms to the theory of optimal foraging [19] and energy maximisation [26].

Furthermore, the results from this study are also in agreement with many others on corallivorous butterflyfish feeding behaviour which all show evidence of selective diets on a sub-set of corals species [5, 8, 28, 29]. However, the nature of these preferences appear to be highly variable among species [28, 30]. Many previous studies have shown butterflyfish favouring *Acropora* species as their primary food source [7, 9, 19, 28], however only one other butterflyfish species, *Chaetodon plebeius*, is reported to have similar preferences to those of *C*. *triangulum* found in this study; selecting for *Pocillopora* in excess of *Acropora* [15]. The reasons for such clear preferences among coral species are still not well understood [15, 23], although many studies assign selectivity to the superior nutritional value and calorific content of certain corals [1, 15, 19]. This notion of selective feeding for energetic benefits cannot fully explain differences among fish however, and it may be factors such as the different morphology of corals and variable jaws/dentition among reef fish which provide a better explanation for interspecific differences [4]. In addition, selectivity has been shown to be visually driven in certain species such as *C*. *baronessa* [31], therefore it is also possible that certain species select, at least in part, for corals whose polyps are extended during daylight hours, greatly reducing the number of corals which can be selected for as many scleractinian polyps only emerge at night. Pratchett [19] suggested it may be that interspecific differences in coral preference have evolved through natural selection, in order to allow coexistence of species on congested coral reefs [15].

*C*. *baronessa* is the eastern sister species of *C*. *triangulum*, replacing it in the Indo-West Pacific with a small overlap in distribution in the Eastern Indian Ocean [32]. Therefore, one could predict that with similar coral assemblage their feeding behaviour would also be similar. Indeed, Berumen [11] showed that *C*. *baronessa*, has similar feeding selectivity and territory size to *C*. *triangulum* illustrated in this study. However, one major difference between the two *Chaetodon* species is that *C*. *baronessa* is frequently reported as being one of the most specialised butterflyfish, favouring *Acropora hyacinthus* over every other available coral. Although the resolution in our study was not detailed enough to recognise more than genus-specific preference, qualitative results of feeding behaviour recognised it was not a single dominant *Acropora* or *Pocillopora* species that was being consumed by *C*. *triangulum*, suggesting that the dietary preferences of *C*. *triangulum* appears to be more conserved than *C*. *baronessa*.

In conclusion, the quantified dietary specialisation of *C*. *triangulum*, ascertained in this study implies that *C*. *triangulum* may be vulnerable if the decline of coral continues at its present rate [33]. *Acropora* and *Pocillopora* species were shown to be the dietary preferences of *C*. *triangulum*, with the latter showing greater preference when available. Incidentally, it is these coral genera, which encompass some of the more susceptible species of scleractinian coral to factors associated with climate change such as increasing sea surface temperatures and therefore bleaching and disease [34]. Therefore, the dietary versatility identified on a small scale in this study may be imperative for the future survival of certain species such as *C*. *triangulum* if such climate change trends continue. Finally, the study adds to the growing list of publications which exemplifies the need for increased efforts of conservation and management in the Maldives, particularly with regard to more manageable local threats such as pollution, over fishing and other tourism impacts such as development, in order to safeguard dependant biota at higher taxonomic levels.
